# Frequency and risk factors of primary sclerosing cholangitis among patients with inflammatory bowel disease in North-East of Iran 

**Published:** 2015

**Authors:** Ahmad Khosravi Khorashad, Mohammad Khajedaluee, Elham Mokhtari Amirmajdi, Ali Bahari, Mohammad Reza Farzanehfar, Mitra Ahadi, Siavash Abedini, Mohammad Reza Abdollahi, Rosita Vakili, Hassan Vossoughi Nia

**Affiliations:** 1 *Department of Internal Medicine, Faculty of Medicine, Mashhad University of Medical Sciences, Ghaem Hospital, Mashhad, Iran*; 2 *Department of Social Medicine, Faculty of Medicine, Mashhad University of Medical Sciences, Mashhad, Iran*; 3 *Gastroenterology and Hepatoloy Department, Nayshabour Faculty of Medical Sciences, Nayshabour, Iran*; 4 *Endoscopic & Minimally Invasive Surgery Research Center, Ghaem Hospital, Faculty of Medicine, MashhadUniversity of Medical Sciences, Mashhad, Iran*; 5 *Center of Pathological and Medical Diagnostic Services, Iranian Academic Center for Education, Culture & Research (ACECR), Mashhad Branch, Mashhad, Iran*

**Keywords:** PSC, IBD, Ulcerative colitis, Crohn’s disease, Prevalence

## Abstract

**Aim:**

To identify primary sclerosing cholangitis (PSC) predisposing factors in order to prevent inflammatory bowel disease (IBD) progression to PSC.

**Background:**

IBD is commonly associated with PSC and there is no effective therapy for PSC except for liver transplantation.

**Patients and methods:**

This retrospective study was conducted on 447 IBD patients from IBD Clinics of Ghaem and Emam Reza Hospitals. Data were collected by interview and through a review of the patients' medical records. Patients were divided into two groups: those with IBD and PSC (IBD-PSC) and those without PSC. Variables were compared between two groups and those with statistically significant differences in IBD-PSC group were considered as predictive factors for the development of PSC.

**Results:**

The frequency of PSC in IBDs was 4.3% and all were ulcerative colitis. The mean age of patients with PSC was 39.1±11.33 years. The male to female proportion in PSCs was 3.8:1 and in IBDs was 0.9:1. There were statistically significant associations between PSC and gender, IBD duration and UC extension, mucocutaneous involvement, oral contraceptive pills (OCP) consumption, history of surgery and history of PSC in the first**- **degree relatives.

**Conclusion:**

PSC frequency among IBD patients in North-East of Iran was 4.3%. It is recommended to limit OCP consumption in IBD patients. Identification and modification of probable predisposing risk factors, as well as early diagnosis of PSC are necessary.

## Introduction

 Inflammatory bowel disease (IBD), chronic inflammatory disorder of the gastrointestinal tract is thought to be the result of inappropriate and mucosal immune over the response to the gut bacteria (1). IBD is generally divided into two major disease entities: ulcerative colitis (UC) and crohn’s disease (CD) (2). UC is the most common form of IBD usually accompanied with chronic bloody diarrhea. In contrast, CD mostly presents with abdominal pain, obstruction and malabsorptive symptoms (3, 4). North American incidence rates for UC range from 2.2-14.3 cases per 100,000 person-years, and from 3.1- 14.6 cases per 100,000 person-years for CD (5). However, environmental, genetic and immunologic factors have been identified as risk factors in the development of IBD disorders; the exact etiology of the disease is still unknown (6). In a study conducted on 566 patients with CD, disease duration over ten years, penetrating of CD, perianal disease and cigarette smoking were the main risk factors for recurrence of CD (7). In addition to intestinal manifestations, IBD is commonly associated with some extra intestinal features and autoimmune disorders such as primary sclerosing cholangitis (PSC) (2, 8). 

PSC is a chronic cholestatic liver disease with uncertain etiology characterized by chronic inflammation and progressive obliterating inflammatory fibrosis of the intra- and extra-hepatic bile ducts which leads to biliary cirrhosis and eventually hepatic failure (9). The most common symptoms of PSC include: fatigue, pruritus, abdominal pain and jaundice, although 15-70% of patients may remain asymptomatic (8, 10, 11). The prevalence of IBD (especially UC) among patients with PSC is about 70–80%, although about 2–7% of patients with UC have PSC (12).

Since PSC is an uncommon disease, the true incidence and prevalence of PSC remains undetermined. In the United States and Norway the incidence and prevalence rates for PSC range from 0.9-1.3 and from 8.5-13.6 per 100,000 person-years, respectively, while the prevalence and incidence of PSC in Southern Europe and Asia seem to be much lower (0.1 per 100,000 a year) (11, 13). The prevalence rate of IBDs in Iran is less than western countries. CD prevalence in the general population of Iran has been reported 0.6-0.96% and the incidence rate for UC were between 3.04% and 3.25% per 10^5^ individuals (14, 15). Both environmental and genetic factors increase the risk of PSC (16). Unlike IBD, PSC has a male predominance (2:1), generally affects children and adults with an average age of onset in the fourth decade, whereas, the association between PSC with IBD and the age of onset vary among populations (9, 17). PSC - similar to UC is more common in non-smokers (11,18). Family history of the disease, previous biliary surgery and cytomegalovirus infection are some other risk factors for development of PSC (10, 16).

Since PSC is a chronic progressive disease, there is no effective treatment except liver transplantation; it would be informative to investigate the probable predisposing factors for development of PSC in patients with IBD. Therefore, the aim of this study was to evaluate the frequency and risk factors related to PSC among patients with IBD.

## Patients and Methods

**Study population**

This retrospective study was conducted on 447 patients with IBD, who were referred to IBD Clinics of Ghaem and Emam Reza University Hospitals during 2011. Of them, 384 (85.9%) had UC and 61 patients (13.6%) had CD. Nineteen IBD patients (4.3%) were diagnosed as having PSC. All of the PSC patients had UC (Table 1). The diagnosis of IBD was based on clinical symptoms, laboratory, as well as histological and radiological findings and confirmed by qualified physicians. Personal and clinical information, as well as risk factors, including demographic information such as age, gender, education and occupation, extension of disease, duration of disease, history of surgery (appendectomy, colectomy, female genital surgeries**, **other), smoking, drug addiction, history of using oral contraceptive pills (OCP), family history of IBD or PSC among first**- **degree relatives, complications related to disease, breastfeeding were collected from patient’s medical recorded files. The diagnosis of PSC was made by ERCP or MRCP, and sometimes with liver biopsy in combination with biochemical findings. Patients were divided into two groups: those with IBD and PSC (IBD-PSC) and those without PSC. Variables were compared between two groups and those with statistically significant differences in IBD-PSC group were considered as predictive factors for development of PSC in patients with IBD. The Research Ethics Committee of Mashhad University of Medical Sciences (MUMS) approved this study (Number: 900437)

**Statistical analysis**

Data on demographic characteristics, clinical and para-clinical features were analyzed by SPSS software version 11.5. Descriptive data were summarized as mean, standard deviation (SD), and percents. Chi-Square test was performed for comparison of qualitative variables between patients with IBD-PSC and patients without PSC. Student’s *t*-tests were used for comparison of quantitative variables if the distributions of the variables were normal and Mann-Whitney test was used if it is assumed that not all data sets meet a normal distribution. The P-value < 0.05 was considered statistically significant. 

## Results

**Demographic characteristics**

The mean age of patients was 38.6±13.34 years (range from 16-83). The male to female proportion in patients with UC was equal; however, CD was more prevalent among women (M/F=0.65). PSC is considerably more common in men (78.9% were male and 21.1 percent were women (3.8:1)). Furthermore, the male to female proportion of patients without PSC was 0.9. Although there was no significant association between gender and IBD, gender was significantly associated with PSC (p=0.007). Moreover, 93.8 % of patients with UC (n=349) and 88.9% of patients with PSC (n=16) were breastfed. There was a statistically significant relationship between breastfeeding and IBD (p=0.003), but this association was not found with PSC patients. UC was more common among unemployed people (30.4%). There was no significant association between IBD and PSC with occupation, education, smoking and opium addiction. 

**Medical conditions**

In this study, 7.2% of patients with UC (n=27) and 23.5% of patients with PSC (n=4) had** a **family history of IBD among their first**- **degree relatives. There was no significant association between family history of IBD among their first**- **degree relatives and PSC, although PSC was significantly associated with a history of PSC among their first**- **degree relatives (p<0.0001). 

Patients with UC showed 45.5% pancolitis (n=166), 36.2% rectosigmoid (n=132) and 18.6% (n=68) rectal involvements. On the other hand, patients with PSC had 94.7% pancolitis and 5.3% proctitis. Moreover, there was a significant relationship between the extension of colonic involvement in UC with PSC (p<0.0001). The mean duration of UC and PSC diseases in IBD patients were 58.6±40.88 and 80.4±27.3 months, respectively. Duration of IBD disease especially UC, was significantly associated with PSC+IBD (p=0.009).

Among the study population, 11.4% of females with UC and 50% of female patients with PSC had a history of OCP consumption. History of OCP consumption and development of the PSC were significantly associated (p=0.01), however development of IBD was not significantly associated with a history of OCP consumption, maybe because of the small sample size.

 History of abdominal surgery and IBD were significantly associated (p<0.0001), but we couldn’t find a statistically significant relationship between PSC and history of surgery. 

Totally, 13.2% of patients with UC and 42.1% of patients with PSC had mucocutaneous involvement. The most common complaint was itching followed by rash and aphthous ulcer. Mucocutaneous involvement and PSC were significantly associated (p<0.0001), however; we couldn’t find any relationship between mucocutaneous involvement and IBD without PSC. Furthermore, 10% of patients were suffering from internal hemorrhoids, including 8.6% of UC patients (n=32) and 5.3% of PSC patients. In addition, there was a statistically significant association between history of internal hemorrhoid disease and IBD (p=0.034). Table 1 shows demographic data, clinical characteristics and risk factors for development of PSC in patients with UC and PSC. 

## Discussion

PSC is a rare chronic disease with unknown etiology, which has been observed in approximately 5% of patients with UC, and it has a lower prevalence in CD. More than 90% of patients with PSC have IBD (18, 19). Liver transplantation is an only effective medical therapy for patients with PSC; therefore in this study, we investigated the frequency and risk factors related to PSC among patients with IBD. The present study demonstrated that the frequency of PSC in patients with IBD was 4.3% and all of the PSC patients had UC. This result is in line with the results of previous studies in the west (16, 18); however, this relatively high prevalence of PSC maybe due to: (i) limited study period, asymptomatic or mildly symptomatic patients did not refer to this subspecialty center, (ii) Or genetic and environmental factors in this region play an important role in developing PSC among patients with IBD. In a study conducted on 200 IBD patients, the occurrence of UC was much higher than CD and IBD was more prevalent in young people with male predominance (20). In the present study, as shown in Table 1, the mean age of patients with PSC and UC were 39.1±11.33 and 38.7±13.4, respectively. We didn’t find a statistically significant relationship between age and IBD or PSC (p=0.76). The age distribution in this study was similar to those of developed countries (21). However, in the present study, the second peak for age distribution was not significant. In the present study PSC was more common in males (M/F=3.8:1), in contrast, IBD was more common in women (M/F=0/9). Furthermore, the gender distribution of cases with UC and PSC–IBD were similar to previous studies (22, 23). There was a statistically significant association between gender and development of PSC in patients with IBD. Male gender was a significant risk factor for PSC (p<0.001). Moreover, in the present study, a significant relationship was found between breastfeeding and development of IBD (p<0.003). We found a statistically significant association between PSC and duration of IBD, especially UC (p<0.001). Previous studies have reported similar results (23, 24). Recent studies have shown that smoking has a protective effect on UC and deleterious effect on CD (4, 19, 25). In the present work, the number of smokers was very low and maybe due to the small sample size, we didn’t find any significant association between smoking and drug abuse with IBD or PSC. On the other hand, due to indecency of drug abuse and smoking in our society, some patients may deny the drug abuse and might gave incorrect information. In addition, consistent with the results of previous studies, we indicated a statistically significant relationship between the extension of UC and development of PSC (p<0.001). The PSC was also more prevalent in patients with pancolitis (24, 26). 

In this study, we find a significant association between history of abdominal surgery and the type of surgery with IBD (p<0.001). Furthermore, in studies conducted in other countries, there was a significant association between appendectomy and development of IBD (18). 

**Table1:** Demographic data, clinical characteristics and risk factors for development of PSC in patients with ulcerative colitis and primary sclerosing cholangitis

**characteristics**	**UC** No (%)	**PSC** No (%)	**P value**
**Number of patients**	384(85.9)	19(4.3)	
**Age**	38.7±13.4	39.1±11.33	0.76
**Gender**			
Female	191(49.7)	4(12.1)	
Male	193(50.3)	15(78.9)	0.007
**Breastfeeding**	349(93.8)	16(88.9)	
**Education**			
Illiterate	13(3.4)	0	
Diploma	188(49.9)	10(52.6)	
Associated diploma	55(14.6)	3(15.8)	0.24
Bachelor	109(28.9)	5(26.3)	
Master and higher	12(3.2)	1(5.3)	
**Occupation**			
Unemployed	117(30.4)	3(15.8)	
Employee	111(28.9)	5(26.3)	
Self-employed	78(20.3)	8(42.1)	0.63
Worker	10(2.6)	0	
Student	68(17.7)	3(15.8)	
**Smoking**	19(5.1)	0	0.30
**Addiction**	9(2.4)	1(5.6)	0.34
**Family history of IBD**			
Sister	8(30.8)	2(50)	
Brother	7(26.9)	2(50)	<0.0001
Mother	7(26.9)	0	
Father	3(11.5)	0	
**Duration of disease**	58.6±40.88	80.4±27.3	0.009
**Extent of disease**			
Pancolitis	166(45.5)	18(94.7)	
Rectosigmoid	132(36.1)	0	<0.0001
Proctitis	68(18.6)	1(5.3)	
**Using OCP**	21(11.4)	2(50)	0.01
**History of surgery**			
**Female genital surgeries**	15(37.5)	0	
Appendectomy	11(27.5)	2(40)	<0.0001
Other	9(22.5)	3(60)	
**Complications related to disease**			
**Muco-cutaneous involvement**	49(13.2)	8(42.1)	
Itching	27(55.1)	7(87.5)	<0.0001
Rash	17(7.34)	1(12.5)	
Aphthous ulcer	5(10.2)	0	
**Internal hemorrhoids**	32(8.6)	1(5.3)	

UC; ulcerative colitis, PSC; primary sclerosing cholangitis

Moreover, family history of the disease has been recognized as another risk factor for development of PSC. The risk of PSC in the first-degree relative is about 0.7% and rise to 1.5% in sibling of patients with PSC (19). In this work, we found statistically significant association between history of PSC in the first-degree relative with IBD (particularly siblings) and development of PSC (p<0.001). Among extra intestinal manifestations of patients with IBD, we only found a significant association between mucocutaneous involvement and PSC (p<0.001). Therefore, mucocutaneous involvement in patients with IBD can be a predictive factor for developing PSC. Also, we observed a statistically significant association between internal hemorrhoids and IBD. Intestinal inflammation probably results in congestion and releasing inflammatory cytokines and factors, which cause vessel dilation especially in distal colon associated with hemorrhoids. In addition, we demonstrated that OCP consumption in women with IBD could be a risk factor for development of PSC (p<0.001). Previous studies reported that OCP consumption contributed to the development of IBD; however, the effect OCP consumption on the development of PSC has not been evaluated yet (27, 28). 

In conclusion, we observed the prevalence of PSC in patients with IBD was similar to other populations. Male gender, duration of IBD, extension of UC, mucocutaneous involvement, history of PSC in the first- degree relatives and OCP consumption were the probable risk factors for development of PSC in IBD patients. Since there is no treatment guideline for the end stage PSC patients except for liver transplantation, prevention, early diagnosis of PSC is necessary. It is also recommended to limit OCP consumption in IBD patients. Further prospective studies with a larger sample size are needed to find other predisposing factors affecting the development of PSC in IBD patients. 

This study was conducted in Khorasan Razavi province; however, further studies are required to determine prevalence and risk factors of PSC in the other parts of the country.

## References

[1] Gaj P, Habior A, Mikula M, Ostrowski J (2008). Lack of evidence for association of primary sclerosing cholangitis and primary biliary cirrhosis with risk alleles for Crohn's disease in Polish patients. BMC Med Genet.

[2] Verdonk RC, Dijkstra G, Haagsma EB, Shostrom VK, Van den Berg AP, Kleibeuker JH (2006). Inflammatory bowel disease after liver transplantation: risk factors for recurrence and de novo disease. Am J Transplant.

[3] Danese S, Fiocchi C (2011). Ulcerative colitis. N Engl J Med.

[4] Hanauer SB, Sandborn W (2001). Management of Crohn's disease in adults. Am J Gastroenterol.

[5] Shanbhogue AK, Prasad SR, Jagirdar J, Takahashi N, Sandrasegaran K, Fazzio RT (2010). Comprehensive update on select immune-mediated gastroenterocolitis syndromes: implications for diagnosis and management. Radiographics.

[6] Safarpour AR, Hosseini SV, Mehrabani D (2013). Epidemiology of Inflammatory Bowel Diseases in Iran and Asia; A Mini Review. Iranian J Med Sci.

[7] Khoshkish S, Arefi K, Charmechali M, Vahedi H, Malekzadeh R (2012). Risk factors for postoperative recurrence of Crohn's disease. Middle East J Dig Dis.

[8] Danese S, Semeraro S, Papa A, Roberto I, Scaldaferri F, Fedeli G (2005). Extraintestinal manifestations in inflammatory bowel disease. World J Gastroenterol.

[9] Aron JH, Bowlus CL (2009). The immunobiology of primary sclerosing cholangitis. Semin Immunopathol.

[10] Silveira MG, Lindor KD (2008). Clinical features and management of primary sclerosing cholangitis. World J Gastroenterol.

[11] Worthington J, Chapman R (2006). Primary sclerosing cholangitis. Orphanet J Rare Dis.

[12] Itou M, Mitsuyama K, Kawaguchi T, Okabe Y, Suga H, Masuda J (2009). Leukocytapheresis therapy improved cholestasis in a patient suffering from primary sclerosing cholangitis with ulcerative colitis. Case Rep Gastroenterol.

[13] Razumilava N, Gores GJ, Lindor KD (2011). Cancer surveillance in patients with primary sclerosing cholangitis. Hepatology (Baltimore, Md).

[14] Tavakkoli H, Haghdani S, Adilipour H, Daghaghzadeh H, Minakari M, Adibi P (2012). Serologic celiac disease in patients with inflammatory bowel disease. J Res Med Sci.

[15] Shayesteh AA, Saberifirozi M, Abedian S, Sebghatollahi V (2013). Epidemiological, demographic, and colonic extension of ulcerative colitis in Iran: a systematic review. Middle East J Dig Dis.

[16] Toy E, Balasubramanian S, Selmi C, Li CS, Bowlus CL (2011). The prevalence, incidence and natural history of primary sclerosing cholangitis in an ethnically diverse population. BMC Gastroenterol.

[17] Silveira MG, Lindor KD (2008). Primary sclerosing cholangitis. Can J Gastroenterol.

[18] Mitchell SA, Thyssen M, Orchard TR, Jewell DP, Fleming KA, Chapman RW (2002). Cigarette smoking, appendectomy, and tonsillectomy as risk factors for the development of primary sclerosing cholangitis: a case control study. Gut.

[19] Saich R, Chapman R (2008). Primary sclerosing cholangitis, autoimmune hepatitis and overlap syndromes in inflammatory bowel disease. World J Gastroenterol.

[20] MasnadiShirazi K, Somi MH, Bafandeh Y, Saremi F, Mylanchy N, Rezaeifar P (2013). Epidemiological and Clinical Characteristics of Inflammatory Bowel Disease in Patients from Northwestern Iran. Middle East J Dig Dis.

[21] Shorbagi A, Bayraktar Y (2008). Primary sclerosing cholangitis-what is the difference between east and west?. World J Gastroenterol.

[22] Fosby B, Karlsen TH, Melum E (2012). Recurrence and rejection in liver transplantation for primary sclerosing cholangitis. World J Gastroenterol.

[23] Moncrief KJ, Savu A, Ma MM, Bain VG, Wong WW, Tandon P (2010). The natural history of inflammatory bowel disease and primary sclerosing cholangitis after liver transplantation-a single-centre experience. Can J Gastroenterol.

[24] Sokol H, Cosnes J, Chazouilleres O, Beaugerie L, Tiret E, Poupon R (2008). Disease activity and cancer risk in inflammatory bowel disease associated with primary sclerosing cholangitis. World J Gastroenterol.

[25] Florin TH, Pandeya N, Radford-Smith GL (2004). Epidemiology of appendicectomy in primary sclerosing cholangitis and ulcerative colitis: its influence on the clinical behaviour of these diseases. Gut.

[26] Jayaram H, Satsangi J, Chapman RW (2001). Increased colorectal neoplasia in chronic ulcerative colitis complicated by primary sclerosing cholangitis: fact or fiction?. Gut.

[27] Cornish JA, Tan E, Simillis C, Clark SK, Teare J, Tekkis PP (2008). The risk of oral contraceptives in the etiology of inflammatory bowel disease: a meta-analysis. Am J Gastroenterol.

[28] Khalili H, Higuchi LM, Ananthakrishnan AN, Richter JM, Feskanich D, Fuchs CS (2013). Oral contraceptives, reproductive factors and risk of inflammatory bowel disease. Gut.

